# 0.5 V, Low-Power Bulk-Driven Current Differencing Transconductance Amplifier

**DOI:** 10.3390/s24216852

**Published:** 2024-10-25

**Authors:** Montree Kumngern, Fabian Khateb, Tomasz Kulej

**Affiliations:** 1 Department of Telecommunications Engineering, School of Engineering, King Mongkut’s Institute of Technology Ladkrabang, Bangkok 10520, Thailand; montree.ku@kmitl.ac.th; 2 Department of Microelectronics, Brno University of Technology, Technická 10, 601 90 Brno, Czech Republic; 3 Department of Electrical Engineering, Brno University of Defence, Kounicova 65, 662 10 Brno, Czech Republic; 4 Department of Electrical Engineering, Czestochowa University of Technology, 42-201 Czestochowa, Poland; kulej@el.pcz.czest.pl

**Keywords:** current differencing transconductance amplifier, bulk-driven MOS transistor, active filter, current-mode circuit, low-voltage low-power circuit

## Abstract

This paper presents a novel low-power low-voltage current differencing transconductance amplifier (CDTA). To achieve a low-voltage low-power CDTA, the BD-MOST (bulk-driven MOS transistor) technique operating in a subthreshold region is used. The proposed CDTA is designed in 0.18 µm CMOS technology, can operate with a supply voltage of 0.5 V, and consumes 1.05 μW of power. The proposed CDTA is used to realize a current-mode universal filter. The filter can realize five standard transfer functions of low-pass, band-pass, high-pass and band-stop, and all-pass from the same circuit. Neither component-matching conditions nor input signals of the inverse type are required to realize these filter functions. The current-mode filter offers low-input and high-output impedance and uses grounded capacitors. The natural frequency and quality factor of the filters can be orthogonally controlled. The proposed CDTA and its applications are simulated using SPICE to confirm the feasibility and functionality of the new circuits.

## 1. Introduction

The current differencing transconductance amplifier (CDTA) is an active building block for current-mode circuit designs that was proposed in 2003 [1]. A standard CDTA has four terminals, two inputs, one output of a differential current signal, and one output current that can be controlled by its transconductance gain. Therefore, this active building block is suitable for current-mode signal processing applications to achieve improved linearity, dynamic range, and reduced power consumption [2]. Many applications of CDTAs have been presented in the literature, i.e., universal filters [3,4,5,6,7,8,9], sinusoidal oscillators [10,11,12,13], wave generators [14,15,16], precision rectifiers [17,18], capacitance multipliers [19,20], inductance simulators [21,22], memristor emulators [23,24,25,26], and chaotic oscillator [27]. It is clear that the CDTA has been exploited extensively for analog signal processing. However, CDTAs that are used in these applications have relatively high power consumption and high supply voltages (i.e., >1 V).

Nowadays, low-voltage and low-power CMOS integrated circuits are in demand for many applications in implantable biomedical electronic systems and the Internet of Things [28,29].

Many CDTA structures using complementary metal-oxide semiconductor (CMOS) and bipolar technologies are available in the open literature [30,31,32,33,34,35,36,37,38,39,40,41,42]. The CDTAs in [30,31] are designed based on a current differencing unit (CDU), using 0.5 μm CMOS technology with a supply voltage of ±2.5 V. The CDTA in [32] improves the input stage to achieve wide bandwidth and low-input impedance (p- and n-terminals), utilizing 0.35 μm CMOS technology with ±1.8 V supply voltage. The CDTA in [33] uses the parasitic resistances at the p- and n-terminals (R_p_ and R_n_), which are useful in circuit designs, and use 0.35 μm CMOS technology with a supply voltage ±1.5 V. An improved CDTA for obtaining input and output stages with high linearity is proposed in [34], and uses 0.35 μm CMOS technology with a supply voltage ±1.5 V. In ref. [35], the input stage of the CDTA is improved to achieve low-input impedance at the p- and n-terminals (<20 Ω). The circuit uses ±1.25 V supply voltage and uses 0.5 μm CMOS technology. A high-performance CDTA for obtaining wide frequency bandwidth and wide linear tuning range of a transconductance amplifier (TA) is introduced in [36], using 0.18 μm CMOS technology with ±0.8 V supply voltage. In ref. [37], a linear tunable transconductor technique was used at the TA output stage to obtain a wide tunable range. The circuit used a supply voltage of ±1.1 V and was used in a field-programmable analog array. In [38,39,40], the CDTA transconductor was improved to achieve high transconductance gain, using 0.18 μm CMOS technology with a supply voltage of 0.9 V. In [41], a CDTA employing the bulk-driven MOS transistor (BD-MOST) technique is proposed, using 0.25 μm CMOS technology with a supply voltage ±0.6 V. It should be noted that the CDTA structures in [30,31,32,33,34,35,36,37,38,39,40] are not designed for operation with supply voltage below 1 V. Among these CDTA structures, the CDTA in [41] uses the lowest supply voltage, i.e., ±0.6 V, which is possible using the bulk-driven MOS transistor (BD-MOST) technique. This technique can overcome the threshold voltage of standard CMOS technologies; thus, low-voltage analog circuits with low power consumption can be achieved. Although several high-performance CDTAs have been introduced, which feature, e.g., wide bandwidth, low-input impedance, and high linearity, making them suitable for high-frequency applications, these CDTA structures [30,31,32,33,34,35,36,37,38,39,40,41] have relatively high power consumption, which is not suitable for low-voltage low-power integrated circuits and low-frequency applications.

In this work, the design of a low-voltage low-power current differencing transconductance amplifier using the BD-MOST technique is proposed. The proposed CDTA is designed in 0.18 µm CMOS technology, uses a supply voltage of 0.5 V, and consumes 1.05 μW of power. The circuit can be used to process low-frequency signals such as biosignals. To confirm that the proposed CDTA can be used for analog signal processing, the circuit was used to realize a current-mode active filter. The filter employs four CDTAs, two grounded capacitors, and two grounded resistors, which can simultaneously realize a low-pass filter (LPF), band-pass filter (BPF), high-pass filter (HPF), band-stop filter (BSF), and all-pass filter (APF) by same circuit by appropriately applying the input signals. The proposed filter possesses low-input and high-output impedances required for current-mode circuits, does not need component-matching conditions or inverse-type input signals, and has the capability of orthogonal and electronic controls of natural frequency and quality factors.

The paper is organized as follows: Section 2 presents the structure of the CDTA using the BD-MOST technique, the proposed current-mode active filters, and the non-ideality analysis. Section 3 shows the simulation results of the CDTA and current-mode active filter. Finally, Section 4 concludes the paper.

## 2. Circuit Description

### 2.1. Proposed Bulk-Driven CDTA

**Figure 1:**
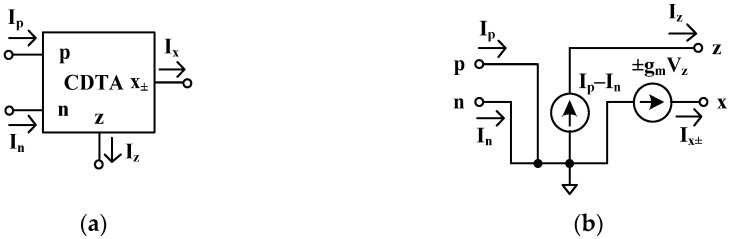
CDTA: (**a**) circuit symbol, (**b**) equivalent circuit.

Figure 1 shows the circuit symbol (a) and equivalent circuit (b) of the CDTA. This device has four terminals, p-, n-, z-, and x-terminals, and its ideal port characteristics can be specified as follows:

(1) $$\left(\begin{array}{c} V_{p}\\ V_{n}\\ I_{z}\\ I_{x\pm } \end{array}\right)=\left(\begin{array}{cccc} 0 & 0 & 0 & 0\\ 0 & 0 & 0 & 0\\ 1 & -1 & 0 & 0\\ 0 & 0 & 0 & g_{m} \end{array}\right)\left(\begin{array}{c} I_{p}\\ I_{n}\\ V_{x}\\ V_{z} \end{array}\right)$$

From Equation (1), the p- and n-terminals are differential input terminals (the p-terminal is the positive current input, and the n-terminal is the negative current input), and the output current of differential input currents appeared at the z-terminal. Thus, $I_{z}=I_{p}-I_{n}$. If the current at the z-terminal is converted to voltage $V_{z}$ using an external impedance load, the voltage $V_{z}$ can be converted to current at the x-terminal ($I_{x}$) using the transconductance $\;g_{m}$ of the CDTA. Thus, $I_{x}$ = $g_{m}V_{z}$. Note that the currents at the p- and n-terminals are the input currents, while the currents at the z- and x-terminals are the output currents; thus, the p- and n-terminals of the CDTA should be low-impedance (zero for ideal), while the z- and x-terminals of the CDTA should be high-impedance (infinite for ideal).

**Figure 2:**
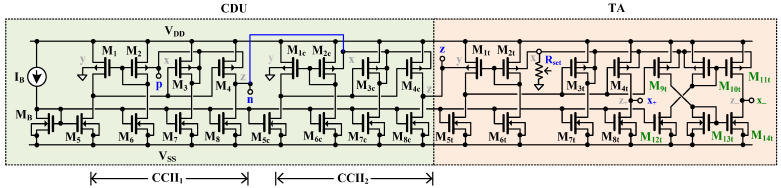
CMOS implementation of proposed 0.5 V CDTA.

Figure 2 shows the CMOS implementation of the proposed CDTA. The circuit consists of a current differencing unit (CDU) followed by a transconductance amplifier (TA). In this work, the principle of creating a CDU using two second-generation current conveyors (CCIIs) is used [43]. The operation of this CDU can be explained as follows: the current $I_{p}$ of the CDTA is applied to the x-terminal of the first CCII_1_, the z-terminal of the first CCII_1_ is connected to the x-terminal of the second CCII_2_, and the current $I_{n}$ of the CDTA is applied to the x-terminal of the second CCII_2_. The z-terminal of the second CCII_2_ is the z-terminal of the CDTA; thus, the current $I_{z}$ of the CDTA can be obtained. Using the CCII’s ideal characteristic, the property $I_{z}=I_{p}-I_{n}$ of the CDTA can be obtained. All y-terminals of both CCIIs are terminated to ground.

Figure 2 shows that the CDU consisted of two bulk-driven second-generation current conveyors (BD-CCII) [44]. The transistors M_1_–M_8_ create the first BD-CCII (CCII_1_), the transistors M_1c_–M_8c_ create the second BD-CCII (CCII_2_). The transistors M_1t_–M_14t_ create the TA, which is also realized using the BD-CCII. The transistor M_B_ and the current I_B_ bias the CDTA. The BD-MOST technique is used to obtain a wide input voltage swing at low supply voltage, while the MOST operating in the subthreshold region is used to obtain a low power consumption of the proposed CDTA.

The current conveyors used in this work are based on precise voltage follower (M_1_–M_3_, M_5_–M_7_) [45], with a doubled output stage for creating a current output of the resulting conveyor. Transistors (M_1_, M_2_), (M_1c_, M_2c_), and (M_1t_, M_2t_) create the non-tailed differential amplifiers loaded with current sources (M_5_, M_6_), (M_5c_, M_6c_), and (M_5t_, M_6t_), respectively. The output voltages of each differential amplifier are respectively applied to the gates of the transistors M_3_, M_3c_, and M_3t_, and these MOSTs are loaded by the current sources M_7_, M_7c_, and M_7t_. The bulk terminals of M_1_ and M_1c_ (Y-terminals of CCII) are connected to the ground. Transistors (M_4_, M_8_), (M_4c_, M_8c_), and (M_4t_, M_8t_) generate copies of the drain currents (M_3_, M_7_), (M_3c_, M_7c_), and (M_3t_, M_7t_); thus, the ideal characteristic $I_{z}=I_{x}$ of the CCII can be obtained [44]. From Figure 2, the x-terminal of the CCII_1_ becomes the p-terminal of the CDTA, the z-terminal of the CCII_1_ is connected to the x-terminal of the CCII_2_ and the n-terminal of the CDTA is connected to this x-terminal of the CCII_2_. The z-terminal of the CCII_2_ becomes the z-terminal of the CDTA. From the principle of the CDU that is explained above, an ideal characteristic of the proposed CDTA can be given as $I_{z}=I_{p}-I_{n}$.

The transistors M_1t_–M_14t_ create the third BD-CCII±, which serves as TA. The $R_{set}$ is an external resistor used to convert voltage to current. From Figure 2, the bulk-terminal of M_1t_ is connected to the z-terminal. When a load is terminated at this z-terminal, the current at the z-terminal ($I_{z}$) is converted to a voltage $V_{z}$. Since the bulk terminals of M_2t_ and M_3t_ are connected to generate unity voltage gain [45], the voltage across R_set_ follows the voltage at V_z_, and this voltage is converted to currents at the x_+_-terminal ($I_{x+}$) and x_−_-terminal ($I_{x-}$). Therefore, the ideal characteristic of $I_{x}=g_{m}V_{z}$ of the CDTA can be obtained. Neglecting the impact of the parasitic resistance of the CCII at the X-terminal, the transconductance $g_{m}$ of the CDTA can be given by

(2) $$g_{m}=\frac{1}{R_{set}}$$

The minus-type x_−_-terminal of the proposed CDTA can be obtained using a cross-coupled current mirror (M_9t_–M_14t_). The multiple-output x_+_- and x_−_-terminals of the CDTA can be obtained using complementary transistor pairs.

Based on the BD-MOST technique and non-tailed differential amplifier, the minimum voltage supply of the proposed CDTA can be obtained as

(3) $$V_{DD(min)}=max\left(V_{SGM2}+V_{DSM6(sat)}\right)$$

The input resistance at the p-terminal ($r_{p}$), the input resistance at n-terminal ($r_{n}$), and the output resistance at the x-terminal of the CDTA ($r_{x}$) can be expressed, respectively, by

(4) $$r_{p}≅\frac{1}{g_{mM3}\frac{g_{mbM1}}{g_{oM1}}}$$

(5) $$r_{n}≅\left.\frac{1}{g_{oM4}+g_{oM8}}\right\|\frac{1}{g_{mM3c}\frac{g_{mbM1c}}{g_{oM1c}}}$$

(6) $$r_{z}≅\frac{1}{g_{oM4c}+g_{oM8c}}$$

(7) $$r_{x}≅\frac{1}{g_{oM4t}+g_{oM8t}}$$

where $g_{mMj}$, $g_{mbMj}$, and $g_{oMj}$ are, respectively, the gate transconductance, bulk transconductance, and output conductance of the jth MOS transistors.

The input referred thermal noise of a non-tailed differential amplifier is used to determine the input noise of the proposed CDTA, which can be expressed by

(8) $${\bar{v}}_{n}^{2}≅\frac{4nkT}{g_{mbM1}^{2}}\left(g_{mM1,M2}+g_{mM5,M6}\right)$$

where n is the subthreshold slope factor, k is the Boltzmann constant (~1.38 × 10^−23^ J/K), and T is the absolute temperature.

### 2.2. Proposed Current-Mode Universal Filter Using CDTA

**Figure 3:**
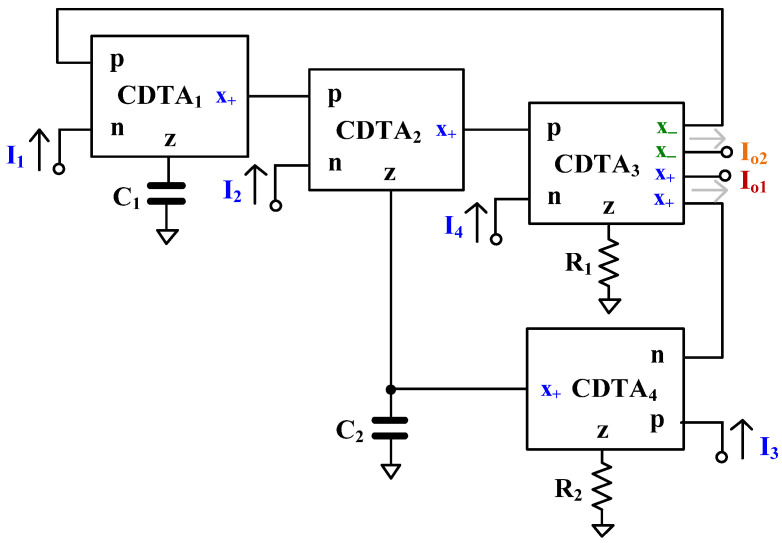
Proposed current-mode universal filter using CDTAs.

Figure 3 shows the proposed current-mode universal filter employing four CDTAs, two grounded capacitors, and two grounded resistors. The $I_{1}$, $I_{2}$, $I_{3}$, and $I_{4}$ are the input currents and the $I_{o1}$ and $I_{o2}$ are the output currents of the circuit. It should be noted that the currents $I_{1}$, $I_{2}$, $I_{3,}$ and $I_{4}$ are applied to the low-impedance terminals of the CDTA and the currents $I_{o1}$ and $I_{o2}$ are obtained from the high-impedance terminals of the CDTA. Therefore, the circuit has low-input impedance and high-output impedance, which is desirable for current-mode circuits. By using grounded passive components, parasitic circuit parameters can be easily compensated.

Using Equation (1) and nodal analysis, the output currents $I_{o1}$ and $I_{o2}$ of the proposed filter can be expressed as

(9) $$I_{o1}=\frac{\left\{\begin{array}{c} -s^{2}C_{1}C_{2}g_{m3}R_{1}I_{4}+sC_{1}g_{m2}g_{m3}g_{m4}R_{1}I_{3}-sC_{1}g_{m2}g_{m3}R_{1}I_{2}\\ -g_{m1}g_{m2}g_{m3}R_{1}I_{1} \end{array}\right\}}{s^{2}C_{1}C_{2}+sC_{1}g_{m2}g_{m3}g_{m4}R_{1}R_{2}+g_{m1}g_{m2}g_{m3}R_{1}}$$

(10) $$I_{o2}=\frac{\left\{\begin{array}{c} s^{2}C_{1}C_{2}g_{m3}R_{1}I_{4}-sC_{1}g_{m2}g_{m3}g_{m4}R_{1}I_{3}+sC_{1}g_{m2}g_{m3}R_{1}I_{2}\\ +g_{m1}g_{m2}g_{m3}R_{1}I_{1} \end{array}\right\}}{s^{2}C_{1}C_{2}+sC_{1}g_{m2}g_{m3}g_{m4}R_{1}R_{2}+g_{m1}g_{m2}g_{m3}R_{1}}$$

where $g_{mj}=1/R_{setj}$ is the transconductance of the jth CDTA (j=1, 2, 3, 4).

From Equations (9) and (10), and using Figure 3, the variant filter responses of the proposed current-mode filter can be obtained by appropriately applying the input signals and selecting the output terminals (the input terminals that are not applied by signals can be floated or connected to ground to avoid external noise injection, while the output signals that are not selected can be neglected).

When the input signal is applied to terminal *I*_1_, the inverting and non-inverting LPF can be obtained at terminals *I_o_*_1_ and *I_o_*_2_, respectively, and the transfer functions can be expressed by Equations (11) and (12), respectively.

(11) $$\frac{I_{o1}}{I_{1}}=-\frac{g_{m1}g_{m2}g_{m3}R_{1}}{s^{2}C_{1}C_{2}+sC_{1}g_{m2}g_{m3}g_{m4}R_{1}R_{2}+g_{m1}g_{m2}g_{m3}R_{1}}I_{1}$$

(12) $$\frac{I_{o2}}{I_{1}}=\frac{g_{m1}g_{m2}g_{m3}R_{1}}{s^{2}C_{1}C_{2}+sC_{1}g_{m2}g_{m3}g_{m4}R_{1}R_{2}+g_{m1}g_{m2}g_{m3}R_{1}}I_{1}$$

When the input signal is applied to terminal I_2_, the inverting and non-inverting BPF can be obtained at terminals *I_o_*_1_ and *I_o_*_2_, respectively, and the transfer functions can be expressed by Equations (13) and (14), respectively. In this case, tuning parameter Q changes the current gain of the transfer functions.

(13) $$\frac{I_{o1}}{I_{2}}=-\frac{sC_{1}g_{m2}g_{m3}R_{1}}{s^{2}C_{1}C_{2}+sC_{1}g_{m2}g_{m3}g_{m4}R_{1}R_{2}+g_{m1}g_{m2}g_{m3}R_{1}}$$

(14) $$\frac{I_{o2}}{I_{2}}=\frac{sC_{1}g_{m2}g_{m3}R_{1}}{s^{2}C_{1}C_{2}+sC_{1}g_{m2}g_{m3}g_{m4}R_{1}R_{2}+g_{m1}g_{m2}g_{m3}R_{1}}$$

When the input signal is applied to terminal *I*_3_, the inverting and non-inverting BPF can be obtained at terminals *I_o_*_1_ and *I_o_*_2_, respectively, and the transfer functions can be expressed by Equations (15) and (16), respectively. In this case, a constant current gain of the transfer function can be obtained by changing the parameter Q.

(15) $$\frac{I_{o1}}{I_{3}}=\frac{sC_{1}g_{m2}g_{m3}g_{m4}R_{1}}{s^{2}C_{1}C_{2}+sC_{1}g_{m2}g_{m3}g_{m4}R_{1}R_{2}+g_{m1}g_{m2}g_{m3}R_{1}}$$

(16) $$\frac{I_{o2}}{I_{3}}=-\frac{sC_{1}g_{m2}g_{m3}g_{m4}R_{1}}{s^{2}C_{1}C_{2}+sC_{1}g_{m2}g_{m3}g_{m4}R_{1}R_{2}+g_{m1}g_{m2}g_{m3}R_{1}}$$

When the input signals (*I*_1_ = *I*_4_ = *I*_in_) are applied to terminals *I*_1_ and *I*_4_, the inverting and non-inverting BSF can be obtained at terminals *I*_o1_ and *I*_o2_, respectively, and the transfer functions can be expressed by Equations (17) and (18), respectively. In this case, circuitry such as the two-output current mirrors are required to provide two identical input signals for applying *I*_1_ and *I*_4_ in Figure 3.

(17) $$\frac{I_{o1}}{I_{in}}=-\left\{\frac{s^{2}C_{1}C_{2}g_{m3}R_{1}+g_{m1}g_{m2}g_{m3}R_{1}}{s^{2}C_{1}C_{2}+sC_{1}g_{m2}g_{m3}g_{m4}R_{1}R_{2}+g_{m1}g_{m2}g_{m3}R_{1}}\right\}$$

(18) $$\frac{I_{o2}}{I_{in}}=\frac{s^{2}C_{1}C_{2}g_{m3}R_{1}+g_{m1}g_{m2}g_{m3}R_{1}}{s^{2}C_{1}C_{2}+sC_{1}g_{m2}g_{m3}g_{m4}R_{1}R_{2}+g_{m1}g_{m2}g_{m3}R_{1}}$$

When the input signals (*I*_1_ = *I*_3_ = *I*_4_ = *I*_in_) are applied to terminals *I*_1_, *I*_3_, and *I*_4_, the inverting and non-inverting BSF can be obtained at terminals *I*_o1_ and *I*_o2_, respectively, and the transfer functions can be expressed, respectively, by Equations (19) and (20). In this case, circuitry such as the three-output current mirrors are required to provide three identical input signals for applying *I*_1_, *I*_3_, and *I*_4_ in Figure 3.

(19) $$\frac{I_{o1}}{I_{in}}=-\left\{\frac{s^{2}C_{1}C_{2}g_{m3}R_{1}-sC_{1}g_{m2}g_{m3}g_{m4}R_{1}+g_{m1}g_{m2}g_{m3}R_{1}}{s^{2}C_{1}C_{2}+sC_{1}g_{m2}g_{m3}g_{m4}R_{1}R_{2}+g_{m1}g_{m2}g_{m3}R_{1}}\right\}$$

(20) $$\frac{I_{o2}}{I_{in}}=\frac{s^{2}C_{1}C_{2}g_{m3}R_{1}-sC_{1}g_{m2}g_{m3}g_{m4}R_{1}+g_{m1}g_{m2}g_{m3}R_{1}}{s^{2}C_{1}C_{2}+sC_{1}g_{m2}g_{m3}g_{m4}R_{1}R_{2}+g_{m1}g_{m2}g_{m3}R_{1}}$$

From Equations (10)–(20), it can be seen that the proposed filter can provide five standard filter responses by appropriate use of input currents. Both non-inverting and inverting transfer functions of LPF, HPF, BPF, BSF, and APF are provided. Thus, the filter topology provides 12 transfer functions in a single topology. It should be noted that the input matching conditions (i.e., $I_{1}=2I_{2}=I_{in}$) and the inverting-type input signals (i.e., $I_{1}=-I_{2}=I_{in}$) are not needed to obtain the variant filter responses.

The natural frequency (ω_o_) and the quality factor (Q) of the filters can be expressed as

(21) $$\omega_{o}=\sqrt{\frac{g_{m1}g_{m2}g_{m3}R_{1}}{C_{1}C_{2}}}$$

(22) $$Q=\frac{1}{g_{m4}R_{2}}\sqrt{\frac{C_{2}g_{m1}}{C_{1}g_{m2}g_{m3}R_{1}}}$$

From Equations (21) and (22), the parameter $\omega_{o}$ can be controlled by $g_{m}$ ($g_{m}=g_{m1}=g_{m2}$) with constant $g_{m3}=1/R_{1}$ and $C_{1}=C_{2}$ while the parameter Q can be controlled by $g_{m4}$ and/or $R_{2}$ with constant $g_{m1}=g_{m2}$, $g_{m3}=1/R_{1}$, and $C_{1}=C_{2}$. The values of these passive and active components can be freely used to control the parameters $\omega_{o}$ and Q.

The non-ideal characteristics of the CDTA have been included to analyze the proposed universal filter. In non-ideal case, the characteristic of the CDTA can be expressed [30,40] by

(23) $$\left.\begin{array}{c} V_{p}=V_{n}=0\\ I_{z}=\alpha_{p}I_{p}-\alpha_{n}I_{n}\\ I_{x}={\beta g}_{m}V_{z} \end{array}\right\}$$

where $\alpha_{p}=1-\varepsilon_{p}$ and $\varepsilon_{p}\left(\left|\varepsilon_{p}\right|\ll 1\right)$ is the parasitic current gain between the p- and z-terminals of the CDTA, $\alpha_{n}=1-\varepsilon_{n}$ and $\varepsilon_{n}\left(\left|\varepsilon_{n}\right|\ll 1\right)$ is the parasitic current gain between the n- and z-terminals of the CDTA, $\beta =1-\varepsilon_{i}$ and $\varepsilon_{i}\left(\left|\varepsilon_{i}\right|\ll 1\right)$ is the output transconductance tracking error from the z- to x-terminals of the CDTA. Ideally, $\alpha_{p}$, $\alpha_{n}$, and β are a unity.

Using Equation (13) and nodal analysis, the denominator of transfer functions can be rewritten as

(24) $$D\left(s\right)=s^{2}C_{1}C_{2}+sC_{1}g_{m2}g_{m3}g_{m4}R_{1}R_{2}\alpha_{p2}\alpha_{p3}\alpha_{n4}\beta_{2}\beta_{3}\beta_{4}\;\;\;\;\;\;\;\;\;\;\;\;+g_{m1}g_{m2}g_{m3}R_{1}\alpha_{p3}\beta_{1}\beta_{2}\beta_{3}$$

In non-ideal case, the natural frequency and quality factor of the proposed filter become

(25) $$\omega_{o}=\sqrt{\frac{g_{m1}g_{m2}g_{m3}R_{1}\alpha_{p3}\beta_{1}\beta_{2}\beta_{3}}{C_{1}C_{2}}}$$

(26) $$Q=\frac{1}{g_{m4}R_{2}{\alpha_{p2}\alpha_{n4}\beta }_{4}}\sqrt{\frac{C_{2}g_{m1}\beta_{1}}{C_{1}g_{m2}g_{m3}R_{1}{\alpha_{p3}\beta }_{2}\beta_{3}}}$$

where $\alpha_{pj}$ and $\alpha_{nj}$ are the parasitic current gains of the jth CDTA and $\beta_{j}$ is the output transconductance tracking error of the jth CDTA.

To avoid the effect of the parasitic capacitances of the CDTA in case the circuit operating at a high frequency, the capacitances $C_{1}$ and $C_{2}$ can be easily selected as $C_{1}>C_{z1}$, $C_{2}>\left.C_{z2}\right\|C_{x4}$, which is the advantage of a circuit with a grounded capacitor, where $C_{zj}$ is the parasitic capacitance at the z-terminal and $C_{xj}$ is the parasitic capacitance at the x-terminal of the jth CDTA.

## 3. Simulation Results

The proposed CDTA is simulated by SPICE with 0.18 μm CMOS parameters. The transistors aspect ratios in µm/µm for the proposed CDTA are M_1_, M_2_, M_1c_, M_2c_, M_1t_, M_2t_, M_B_ = 50/1, M_3_, M_4_, M_3c_, M_4c_, M_3t_, M_4t_, M_9t_, M_10t_, M_11t_ = 5 × 50/1, M_5_, M_6_, M_5c_, M_6c_, M_5t_, M_6t_ = 100/1, and M_7_, M_8_, M_7c_, M_8c_, M_7t_, M_8t_, M_12t_, M_13t_, M_14t_ = 5 × 100/1. The supply voltage is 0.5 V (for the purpose of simulation V_DD_ = −V_SS_ = 0.25 V), and the bias current (I_B_) is 20 nA. It is worth mentioning that the standalone BD-CCII was introduced and experimentally verified in [44]. The extensive post-layout simulation results of process, voltage, temperature (PVT) corners, and Monte Carlo (process and mismatch) analysis confirmed the functionality of the BD-CCII circuit even for a voltage supply of 0.3 V [44].

**Figure 4:**
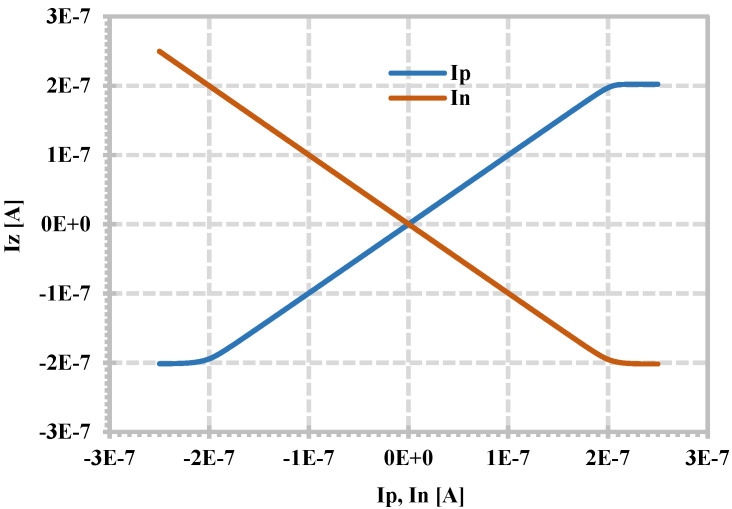
Simulated DC characteristics I_z_ versus I_p_, I_n_ for V_z_ = 0.

Figure 4 shows the simulated DC characteristics of I_z_ versus I_p_ and I_n_ when the z-terminal is grounded, which can be used to show the characteristic of the CDU of the proposed CDTA. The simulation results show that the linear range of the input currents I_p_, I_n,_ and the output current I_z_ is 200 nA.

**Figure 5:**
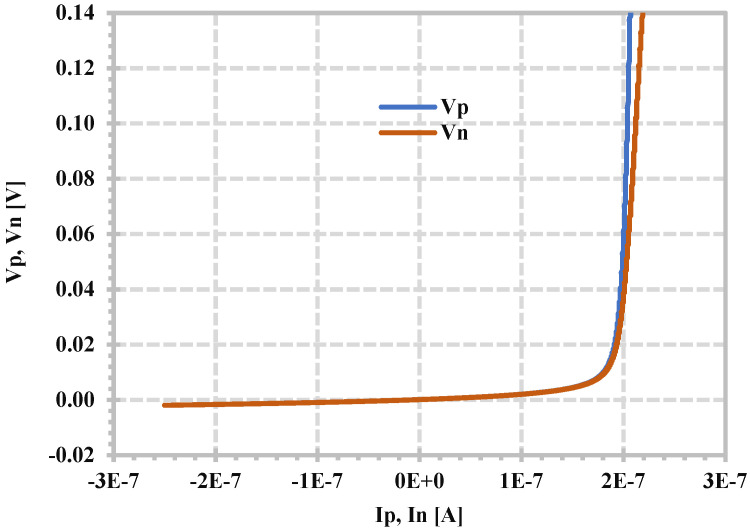
Simulated DC characteristics V_p_ against I_p_ and V_n_ against I_n_.

Figure 5 shows the voltage–current characteristics of the p- and n-terminals when the z-terminal is grounded, which can be used to confirm the linear range of 200 nA of the proposed CDTA. At the input current of 200 nA, the voltage error between V_p_ and V_n_ is about 40 mV.

**Figure 6:**
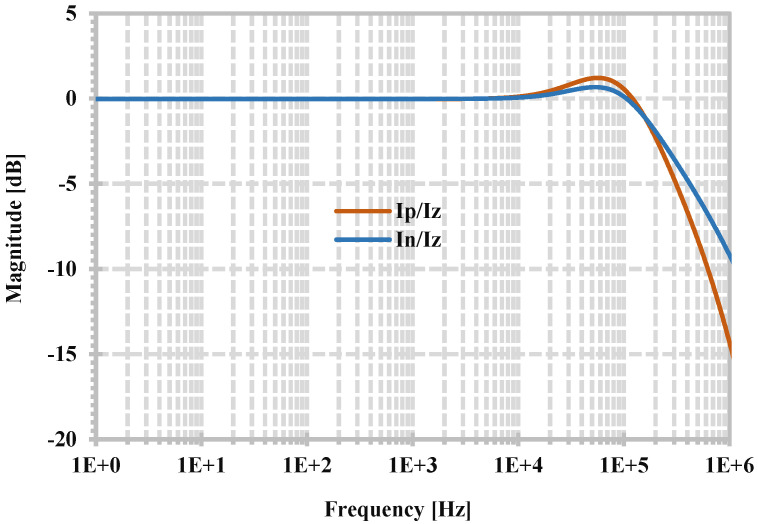
Simulated frequency responses of current gains I_z_/I_p_ and I_z_/I_n_.

Figure 6 shows the simulated frequency responses of the current gains I_z_/I_p_ and I_z_/I_n_ of the proposed CDTA (when the z-terminal is grounded). Compared with (13), the corresponding small-signal current gains are α_p_ = I_z_/I_p_ = 0.996, α_n_ = I_z_/I_n_ = 0.997, and at −3 dB, the bandwidth of the gains α_p_ and α_n_ are 225 kHz and 261 kHz, respectively.

**Figure 7:**
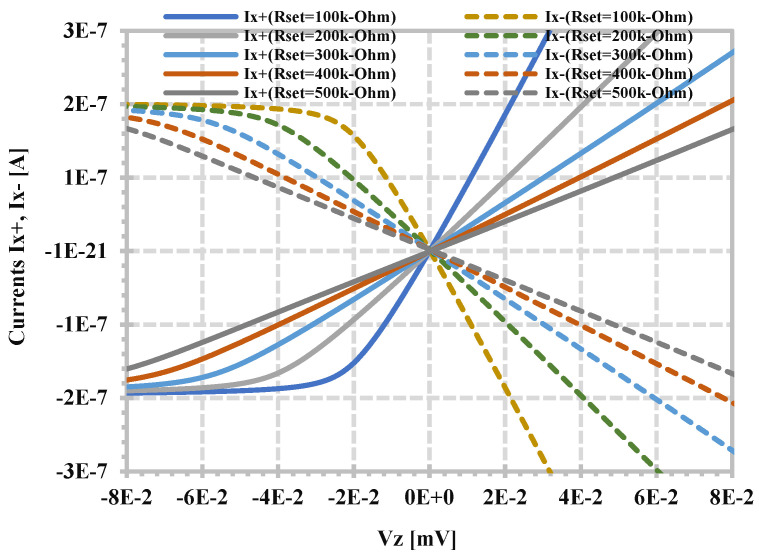
Simulated DC characteristics of TA when R_set_ is varied.

Figure 7 shows the DC characteristic of the I_x+_, I_x−_ versus V_z_ of the proposed CDTA when x+- and x_−_-terminals are grounded and for different values of R_set_, which can confirm that the transconductance can be controlled via external resistance R_set_.

**Figure 8:**
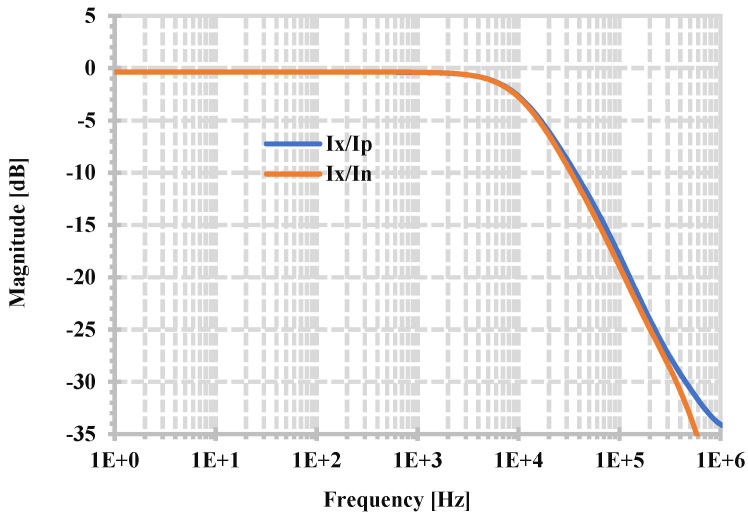
Frequency responses of the current gains I_x_/I_p_ and I_x_/I_n_ (R_z_ = R_set_ = 300 kΩ).

Figure 8 shows the overall simulated frequency range of the proposed CDTA when R_set_ is 300 kΩ, and the z-terminal is terminated with a load of 300 kΩ (R_z_). The 3-dB bandwidths of the current gains I_p_/I_x_ and I_n_/I_x_ are 11.86 kHz and 11.54 kHz, respectively, which is sufficient for bio-signal applications.

**Figure 9:**
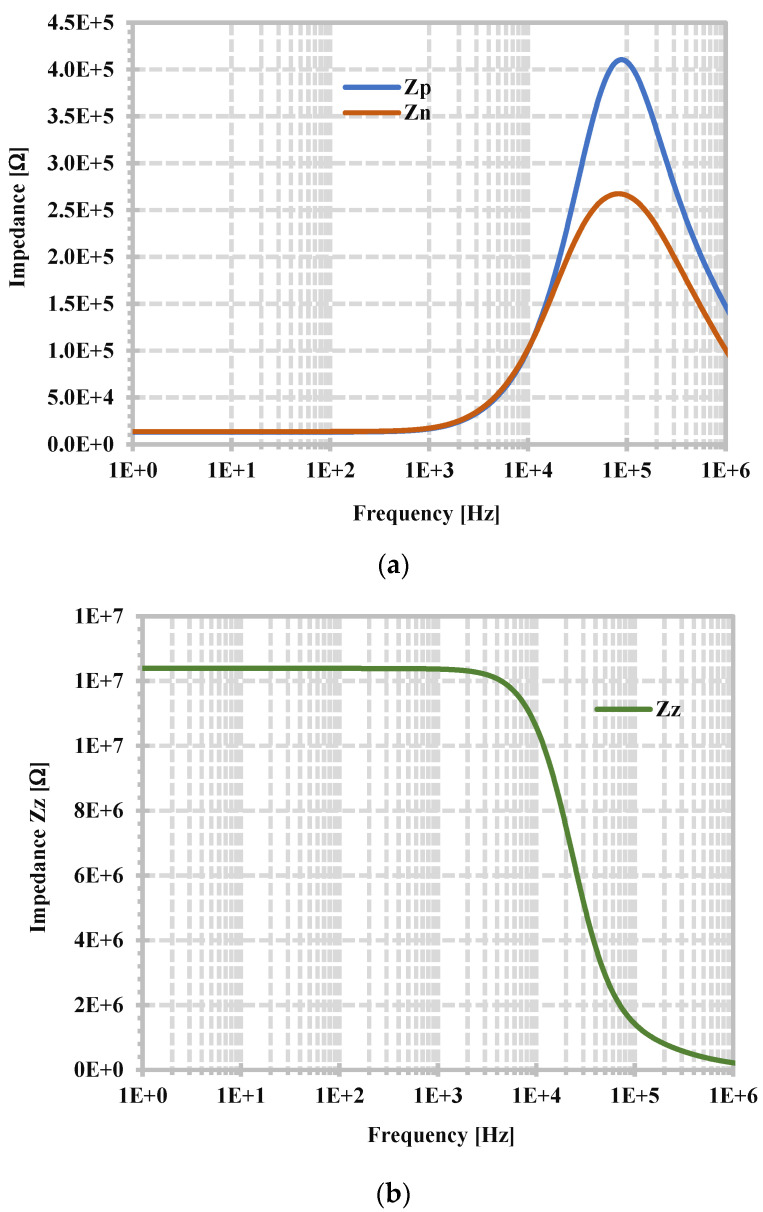
Frequency dependence of the impedances of (**a**) p- and n-terminals, (**b**) z-terminal.

Figure 9 shows the simulated frequency dependence of the parasitic impedances of p-, n-, and z-terminals of the proposed CDTA by applying 1 AC current signal to a specific terminal while unused terminals are grounded, then Z = V_in_/1 = V_in_. From Figure 9a,b, the parasitic resistance of p- and n-terminals (R_p_, R_n_) are, respectively, 13.13 kΩ and 13.08 kΩ, and the parasitic resistance of z-terminal (R_z_) is 12.29 MΩ. The summarized performance of the proposed CDTA is shown in Table 1.

**Figure 10:**
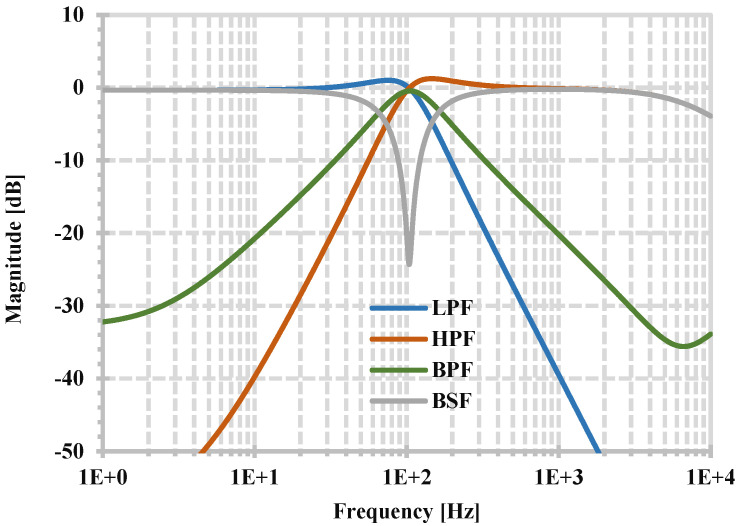
Simulated magnitude frequency responses of LPF, HPF, BPF, and BSF.

**Figure 11:**
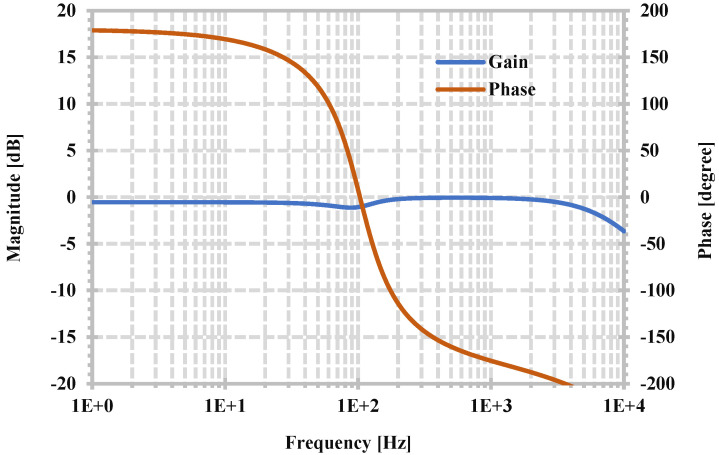
Simulated magnitude and phase frequency responses of APF.

The proposed current-mode universal filter using the proposed CDTA has been simulated. The capacitances C_1_ and C_2_ of 5 nF are used. These capacitors can be realized off-chip for integrated circuit implementation. The filtering functions obtained from the output I_o1_ are selected to be shown. Figure 10 shows the simulated magnitude frequency responses of LPF, HPF, BPF, and BSF when the circuit is designed using R_set1,2,3,4_ = R_1,2_ = 300 kΩ, and Figure 11 shows the simulated magnitude and phase frequency responses of the APF. These simulation results confirm that the proposed filter can provide five standard filter responses. The filter has a natural frequency of 104.7 Hz and consumes 3.39 μW of power, whereas the theoretical value of the natural frequency is 106.1 Hz.

**Figure 12:**
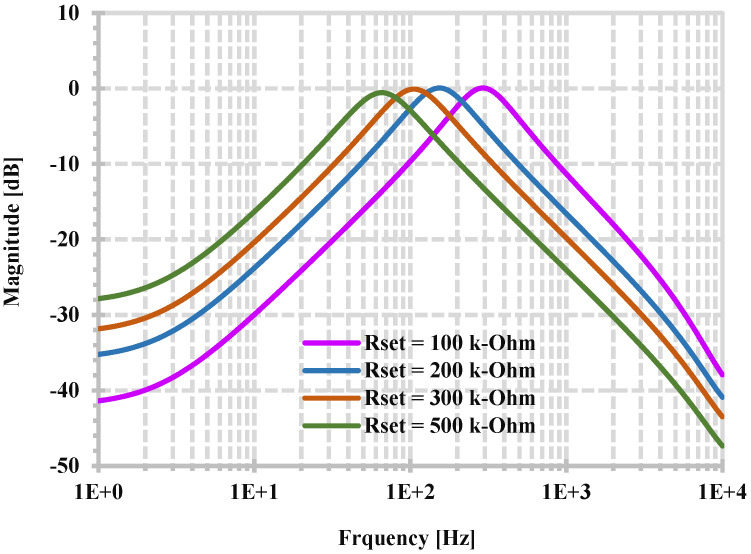
Simulated frequency responses of BPF when the resistors R_set_ (R_set_ = R_set1_ = R_set2_) are changed.

Figure 12 shows the simulated frequency response of BPF when the R_set1_ and R_set2_ are changed (R_set1_ = R_set2_ = R_set_) while the R_set3,4_ and R_1,2_ are fixed. In this case, the input I_3_ is applied as the input signal because a constant current gain can be easily obtained. When the resistors R_set_ are changed from [100, 200, 300, 400] kΩ, the natural frequencies are, respectively, [291.74, 153.1, 104.71, 65.31] Hz.

**Figure 13:**
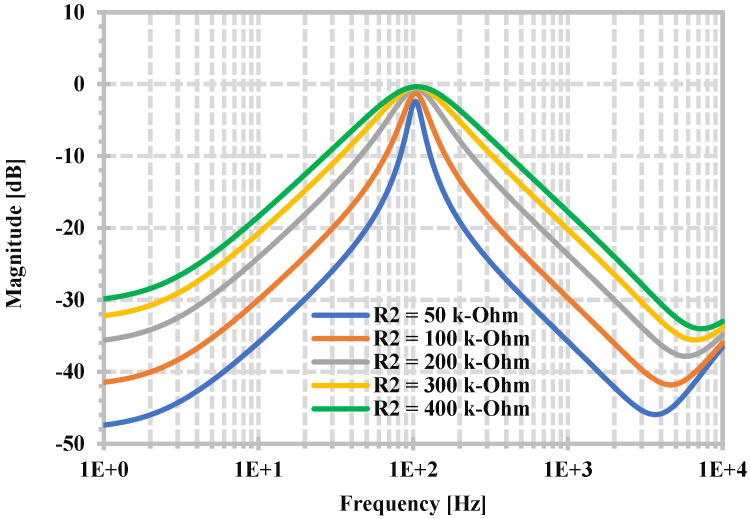
Simulated frequency responses of BPF when R_2_ is varied.

To demonstrate the independent tuning of the Q filter, Figure 13 shows the frequency characteristics of the BPF when the R_2_ is changed to [50, 100, 200, 300, 400] kΩ while R_set1,2,3,4_ and R_1_ are fixed as 300 kΩ.

**Figure 14:**
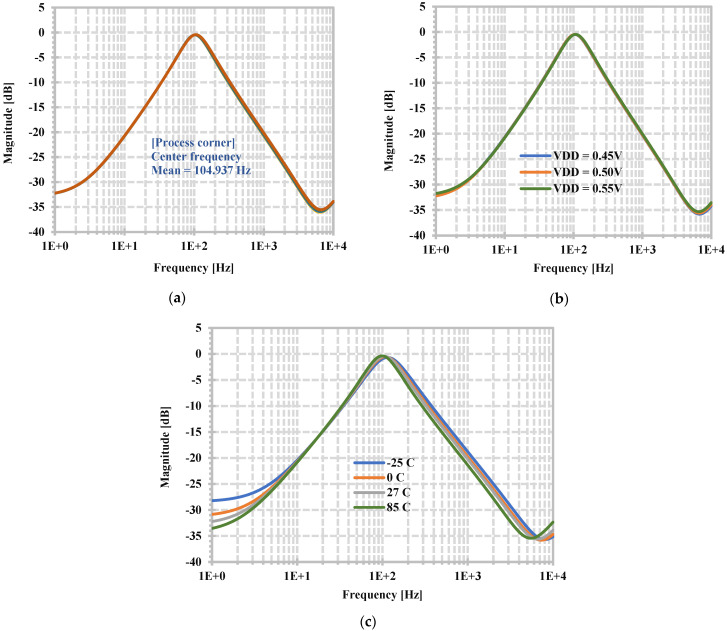
Simulated magnitude frequency response of the BPF when the parameters: (**a**) process, (**b**) supply voltage, and (**c**) temperature are varied.

Figure 14 shows the simulated magnitude frequency responses of the BPF when the parameters of process, voltage, and temperature (PVT) are varied. Figure 14a shows the simulated magnitude frequency responses of the BPF when the threshold voltage in the CMOS process is varied by 5% (LOT tolerance). In this case, Monte Carlo analysis with 200 runs is used for simulation, and 50 simulated magnitude frequency responses are selected to be shown. This simulation result is used to show the impact of process variation.

Figure 14b shows the magnitude frequency responses when the supply voltage is varied by ±10% (0.45 V, 0.5 V, 0.55 V). Figure 14c shows the magnitude frequency responses when the temperature is set to −25, 0, 27, and 85 °C. In this case, when the temperature is changed from −25, 0, 27, and 85 °C, the natural frequencies are, respectively, 114.8, 108.39, 104.7, and 98.8 Hz. Thus, from Figure 14, the filter robustness under PVT variations is confirmed.

**Figure 15:**
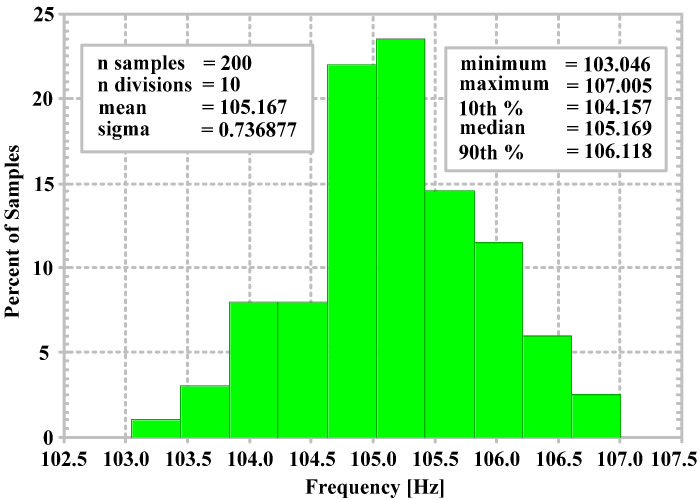
Simulated Monte Carlo analysis for the BPF with 1% tolerance of the capacitors $C_{1}$ and $C_{2}$.

The sensitivity of the capacitors $C_{1}$ and $C_{2}$ on the proposed filter is investigated. The BPF is selected to be tested by using the Monte Carlo analysis with 200 runs and 1% tolerance (LOT) of the capacitors $C_{1}$ and $C_{2}$. Figure 15 shows the histogram of the Monte Carlo analysis of the center frequency for the BPF. From Figure 15, the standard deviation (sigma) is 0.736 Hz, the mean is 105.167 Hz, and the minimal and maximal center frequencies are 103.046 Hz and 107.0 Hz, respectively.

**Figure 16:**
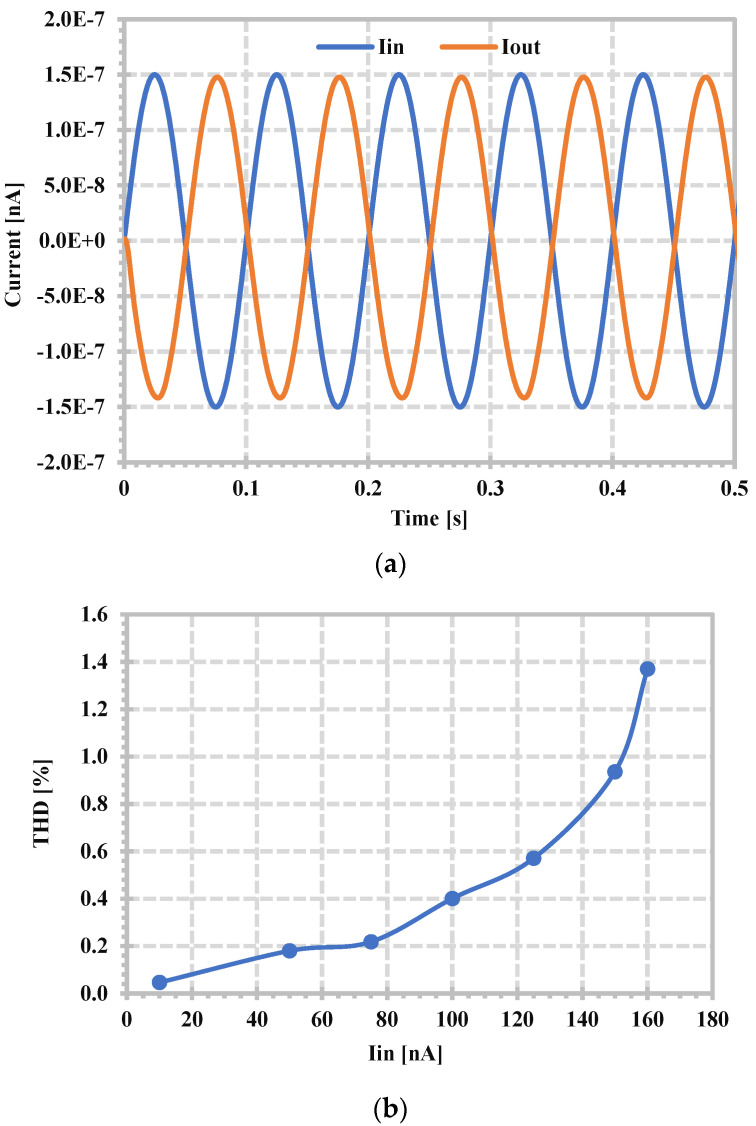
Simulated THD of the LPF, (**a**) input and output waveforms of 0.936% THD, (**b**) dependence of THD on the input signal magnitude.

**Figure 17:**
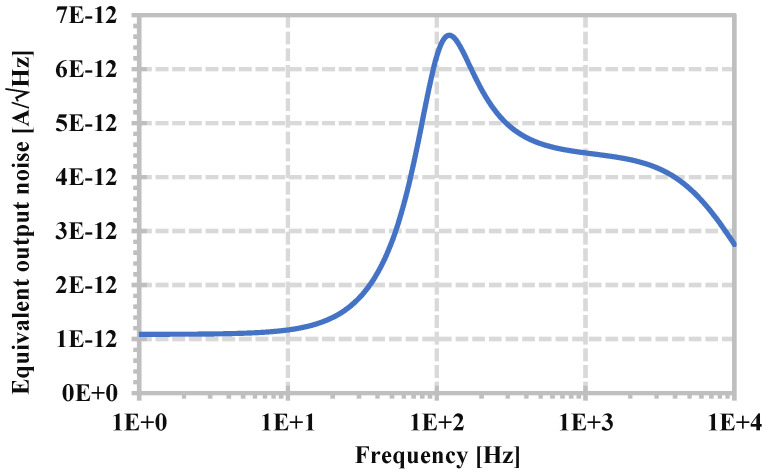
The equivalent output noise of the LPF.

The linearity of the proposed filter is investigated using a single-tone test. The LPF with the 104.7 Hz cut-off frequency was selected, and the in-band input frequency of 10 Hz was applied. The input and output waveforms of 150 nA for 0.936% of total harmonic distortion (THD) are shown in Figure 16a,b shows the overall THD versus the amplitude of the input signal. The equivalent output current noise of the LPF is shown in Figure 17. The integrated output noise in the 104.7 Hz bandwidth was calculated to be 38.32 pA, giving a dynamic range of 68.86 dB.

The proposed current-mode universal filter is compared with previous CDTA-based filters in [4,5,34,39,46,47], as shown in Table 2. It is clear that the proposed filter offers a maximum number of filtering responses, low supply voltage, and low power consumption compared to these previous filters. The filter in [4] uses two CDTAs and offers five standard filtering functions, but inverted/double input conditions are required, while the filters using two CDTAs in [34,39] offer only three filtering functions of LPF, HPF, BPF, and some output currents are supplied through capacitors that require buffer circuits. The filter in [47] using two CDTAs is designed for use in biomedical signal processing, but inverted/double input conditions are required, and the parameters $\omega_{o}$ and Q cannot be orthogonally controlled. The filters in [5,46] have the same number of active CDTAs compared to the proposed filter, but the filter in [5] offers five filter responses, and the filter in [46] offers three filtering responses, while the proposed filter offers twelve filtering responses of both non-inverting and inverting transfer functions of LPF, HPF, BPF, BSP, and APF.

## 4. Conclusions

In this paper, a 0.5 V, low-power bulk-driven current differencing transconductance amplifier (CDTA) is presented. Using a bulk-driven MOS transistor technique-based non-tailed differential amplifier, the proposed CDTA can operate with a 0.5 V supply voltage. The proposed CDTA is designed in a 0.18 µm CMOS technology and consumes 1.05 μW of power. To show the advantage of the proposed CDTA, it has been used to realize a current-mode universal filter. The proposed filter offers the advantages of (i) realization of non-inverting and inverting transfer functions of low-pass, band-pass, high-pass, band-stop, and all-pass filters into the same circuit, (ii) realizing these filtering functions, absent from component-matching conditions and inverting-type input signals, (iii) orthogonal and electronic control of parameters ω_o_ and Q, (iv) low-input and high-output impedances that are required for current-mode circuits, (v) easy to compensate parasitic capacitance when grounded capacitors are used. SPICE simulations demonstrated the effectiveness of the proposed CDTA and its application. The proposed CDTA circuit can be used to process low-frequency signals, such as biosignals, which require low-voltage and low-power circuits.

## Figures and Tables

**Table 1:** Performance data of the proposed CDTA.

Parameters	Value
Supply voltage	0.5 V
Bias current I_B_	20 nA
Technology	0.18 μm
DC current swings: I_z_/I_p_, I_z_/I_n_	±200 nA
Current gains: I_z_/I_p_, I_z_/I_n_	0.996, 0.997
Offset current I_z_ (I_p_ = I_n_ = 0)	0.16 nA
−3 dB bandwidth: I_z_/I_p_, I_z_/I_n_	225, 261 kHz
−3 dB bandwidth of g_m_ (R_z_ = R_set_ = 300 k) I_p_/I_x+_, I_n_/I_x+_, I_p_/I_x−_, I_n_/I_x−_	[11.86, 11.54, 11.54, 11.24] kHz
Z_p_, Z_n_	13.13 kΩ, 13.08 kΩ
Z_z_	12.29 MΩ
Z_x+_, Z_x−_	[12.39, 13.01] MΩ
g_m,set_ (R_set_ = 300 k)	3.28 μA/V
Power dissipation	1.05 μW

**Table 2:** Comparison of the proposed current-mode universal filter with those of some previous filters.

Factor	Proposed 2024	[4] 2007	[5] 2011	[34] 2011	[39] 2022	[46] 2022	[47] 2020
Number of active devices	4-CDTA	2-CDTA	4-CDTA	2-CDTA	2-CDTA	4-CDTA	2-CDTA
Realization	0.18 µm CMOS	BJT process (ALA400 CBIC-R)	0.35 µm CMOS	0.35 µm CMOS	0.18 µm CMOS	90 nm EKV MOSFET	0.18 µm CMOS
Number of passive devices	2-C, 2-R	2-C	2-C, 2-R	2-C	2-C	2-C	2-C
Total number of offered responses	12	5	5	3	3	3	5
$\mathrm{Electronic}\;\mathrm{control}\;\mathrm{of}\;\omega_{o}$	Yes	Yes	Yes	Yes	Yes	Yes	Yes
$\mathrm{Orthogonal}\;\mathrm{control}\;\mathrm{of}\;\omega_{o}$ and Q	Yes	No	Yes	No	Yes	Yes	No
All passive devices grounded	Yes	Yes	Yes	No	Yes	Yes	No
Without inverted/double input conditions	Yes	No	Yes	Yes	Yes	Yes	No
Low-input impedance	Yes	Yes	Yes	Yes	Yes	Yes	Yes
High-output impedance	Yes	Yes	Yes	No	No	Yes	Yes
Power supply (V)	0.5	±3	±1.8	±1.5	±0.9	±1.2	±0.8
Power dissipation (μW)	3.39	-	19 × 10^3^	-	13 × 10^3^	6.34 × 10^3^	-
Natural frequency (Hz)	104.7	318 × 10^3^	1.4 × 10^6^	10.7 × 10^6^	4.82 × 10^6^	903.56 × 10^6^	20.96
THD (%)	0.936@150 nA	-	<4@200 μA (BPF)	-	-	0.089@1 μA (BPF)	-
Dynamic range (dB)	68.86	-	-	-	-	-	-
Verification of result	Sim	Sim	Sim	Sim	Sim	Sim	Sim

## Data Availability

Data are contained within the article.
